# Development and validation of an immune gene-set based prognostic signature for soft tissue sarcoma

**DOI:** 10.1186/s12885-021-07852-2

**Published:** 2021-02-08

**Authors:** Rui Shen, Bo Liu, Xuesen Li, Tengbo Yu, Kuishuai Xu, Jinfeng Ma

**Affiliations:** 1 Department of Spinal Surgery, the Affiliated Hospital of Qingdao University, Qingdao, 266000 China; 2 Department of Sport Medicine, The Affiliated Hospital of Qingdao University, Qingdao, 266000 China

**Keywords:** Soft tissue sarcoma, Immune-related gene, Nomogram, Immune infiltration

## Abstract

**Background:**

Sarcomas is a group of heterogeneous malignant tumors originated from mesenchymal tissue and different types of sarcomas have disparate outcomes. The present study aims to identify the prognostic value of immune-related genes (IRGs) in sarcoma and establish a prognostic signature based on IRGs.

**Methods:**

We collected the expression profile and clinical information of 255 soft tissue sarcoma samples from The Cancer Genome Atlas (TCGA) database and 2498 IRGs from the ImmPort database. The LASSO algorithm and Cox regression analysis were used to identify the best candidate genes and construct a signature. The prognostic ability of the signature was evaluated by ROC curves and Kaplan-Meier survival curves and validated in an independent cohort. Besides, a nomogram based on the IRGs and independent prognostic clinical variables was developed.

**Results:**

A total of 19 IRGs were incorporated into the signature. In the training cohort, the AUC values of signature at 1-, 2-, and 3-years were 0.938, 0.937 and 0.935, respectively. The Kaplan-Meier survival curve indicated that high-risk patients were significantly worse prognosis (*P* < 0.001). In the validation cohort, the AUC values of signature at 1-, 2-, and 3-years were 0.730, 0.717 and 0.647, respectively. The Kaplan-Meier survival curve also showed significant distinct survival outcome between two risk groups. Furthermore, a nomogram based on the signature and four prognostic variables showed great accuracy in whole sarcoma patients and subgroup analyses. More importantly, the results of the TF regulatory network and immune infiltration analysis revealed the potential molecular mechanism of IRGs.

**Conclusions:**

In general, we identified and validated an IRG-based signature, which can be used as an independent prognostic signature in evaluating the prognosis of sarcoma patients and provide potential novel immunotherapy targets.

**Supplementary Information:**

The online version contains supplementary material available at 10.1186/s12885-021-07852-2.

## Background

Sarcoma is a rare group of heterogeneous malignant tumors originated from mesenchymal tissue, mostly occurring in the soft tissue and ending of the long bone [1]. Sarcoma can be classified into more than 80 histological types according to the histological and molecular features [2]. Osteosarcoma, leiomyoma, lymphosarcoma, and synovial sarcoma are four common histological types [3]. Although sarcoma only accounts for 1% of malignancies, they account for 10% ~ 12% of malignancies in children and adolescents [2, 4, 5]. The incidence in recent years is 2.49 ~ 5.87 per person-year [6–9] and the 5-years survival rate after diagnosis was 56.4% ~ 61.6% [6–8]. However, 40% ~ 50% of sarcoma patients could develop metastasis [3, 10], which makes it difficult to choose an appropriate treatment, such as surgery, chemotherapy, and radiotherapy. Therefore, it’s important to find effective makers for risk assessment for sarcoma patients.

Recently, a number of markers, including alternative splicing events, lncRNA, and miRNA, have been identified as prognostic biomarkers for sarcoma patients [11–14]. Unfortunately, these potential biomarkers have been unable to be used in clinical practice, which may be caused by the weak prognostic ability and lacking validation. Lately, a large number of studies were performed to investigate the role of immune-related features in malignant tumors. Changes in the immune system have been shown to play an important role in tumorigenesis and development [15]. For this, immunotherapy is considered as a new and powerful therapy, especially for targeting programmed cell death 1(PD1) and Programmed Death Ligand-1(PDL-1) techniques. By acting on the immune checkpoints, it has been used to treat a variety of cancers, including sarcoma [16]. More importantly, previous studies indicated that immune-related genes (IRGs) can serve as an effective prognostic biomarkers in many tumors, such as urological cancer [17, 18], digestive cancer [19–22] and non–small cell lung cancer [23–26]. Nonetheless, the role of IRGs in sarcoma remains unknown but urgent.

Therefore, the aim of our study was to study the prognostic value of IRGs in sarcoma cohort based on the RNA-sequencing and clinical data from TCGA-SARC (https://cancergenome.nih.gov/) and the IRGs from ImmPort database (https://www.immport.org/shared/). In addition, the regulatory network between prognostic transcription factors (TFs) and prognostic IRGs was established. Finally, a nomogram based on the IRGs and prognostic clinical variables was developed and evaluated.

## Methods

### Patient samples and IRGs

RNA-sequencing (RNA-seq) expression profile and corresponding clinical information of soft tissue sarcoma patients were downloaded from TCGA database, and the clinical information of all patients are shown in Table 1. Meanwhile, the IRG set was obtained from the ImmPort database, which covered 2498 genes, including antigen processing and presentation, antimicrobials, B cell antigen receptor (BCR) signaling pathway, chemokines, chemokine receptors, etc. Then, 170 patients (2/3) were randomly selected to form the training set and the remaining 85 patients (1/3) were incorporated into the testing set.

**Table 1:** Clinical characteristics of sarcoma patients

	Training set	Validation set	t/X^2^	P
Age	60.74 ± 15.29	60.46 ± 13.50	0.141	0.888
Sex			0.198	0.657
Male	79	37		
Female	91	48		
Race			1.074	0.783
Asian	5	1		
African American	11	7		
WHITE	149	75		
NA	5	2		
Histological type			4.753	0.314
LMS	65	37		
DLP	34	23		
UPS	35	14		
MYX	18	7		
Other	18	4		
Metastasis			1.282	0.527
No	84	36		
Yes	34	21		
NA	52	28		
Surgical margin resection status			0.301	0.860
R0	104	49		
R1–2	49	27		
NA	17	9		
Radiotherapy			0.672	0.715
No	94	43		
Yes	46	27		
NA	30	15		
Multifocal indicator			0.699	0.705
No	131	64		
Yes	24	15		
NA	15	6		

*LMS* Leiomyosarcoma, *DLP* Dedifferentiated liposarcoma, *UPS* Undifferentiated pleomorphic sarcoma, *MYX* Myxofibrosarcoma

### Identification of OS-related IRGs in sarcoma patients and enrichment analysis

To identify the prognostic value of IRGs in sarcoma patients, we performed the univariate Cox proportional hazard model to identify the overall survival (OS)-related IRGs in the training set, and genes with a *p* < 0.05 were considered as the OS-related genes. Meanwhile, to further understand the function of OS-related IRGs, Gene Ontology (GO), including molecular function (MF), biological process (BP), and cellular component (CC) and Kyoto Encyclopedia of Genes and Genomes (KEGG) database were used for enrichment analysis. The GO and KEGG analyses were performed by the R package clusterprofiler [27].

### Construction of a transcription factor regulatory network

To determine the possible mechanisms behind the regulation of OS-related IRGs in soft tissue sarcoma patients, we analyzed the correlation between the expression profile data of OS-related TFs and OS-related IRGs. TF set was downloaded from the Cistrome Cancer (http://cistrome.org/). Then, we used univariate Cox proportional hazard model to detect OS-related TFs. The correlation between OS-related TFs and OS-related IRGs were performed by the Pearson correlation analysis. Correlation with a *p* < 0.01 and *r* > 0.5 was considered to be statistical significance. To better illustrate regulatory relationships between the TFs and IRGs, TF-based regulatory network was generated by Cytoscape [28].

### Construction and evaluation of IRGs prognostic signature

Based on the OS-related IRGs identified in the univariate Cox proportional hazard model, we performed the LASSO analysis to avoid overfitting [29]. Then, the significant genes in the LASSO regression were incorporated into the multivariate Cox analysis to detect the ultimate prognostic IRGs of the signature. A prognostic signature was established based on ultimate prognostic IRGs of the signature and the risk score was acquired according to the following formula:

$$ Risk\ \mathrm{Score}=\sum \limits_{i=0}^n{\beta}_i\ast {\mathrm{G}}_i $$

Here, ‘*G*_*i*_’ is the expression of the selected gene, and ‘*β*_*i*_’ is the coefficient of the gene from the multivariate Cox proportional hazards analysis.

To identify the prognostic ability of the IRGs-based signature, time-dependent receiver operating characteristic (ROC) curves were generated, and the corresponding time-dependent area under the curve (AUC) was calculated simultaneously. In addition, 170 patients were stratified into the high- and low-risk group based on the median of risk score. Kaplan-Meier (K-M) survival curve and log-rank test was performed to show the difference of survival status between two groups [30].

### External validation of IRGs prognostic signature

According to the prognostic signature developed in the training set, risk scores of 85 patients in the testing set were calculated. and all patients were classified into high- and low-risk groups. Similarly, K-M survival curves with log-rank test and ROC analysis were utilized to evaluate the accuracy of the prognostic signature in an independent validation cohort.

### Development of a nomogram based on IRGs and clinical variables

In the present study, the data of age, sex, race, histological type of tumor, tumor site and patient metastatic status were obtained from TCGA portal. To investigate whether the risk model is independently associated with the prognosis of soft tissue sarcoma patients, univariate Cox analysis was performed to identify the prognostic variables, which were incorporated into the multivariate Cox analysis to determine the independent prognostic factors. Furthermore, based on the independent prognostic factors, a prognostic nomogram was established [31]. C-index, calibration curve, and decision curve analysis (DCA) were used to evaluate the performance of the nomogram [32].

### Identification of immune infiltration cell in low- and high-risk group

Previous studies showed that IRGs can influence the immune cell composition of tumor microenvironment. Therefore, to further understand the difference of immune infiltration cell between low- and high-risk groups, CIBERSORT package was used to calculate the 22 types of immune cells for each sample, and only samples with CIBERSORT *p* < 0.05 were included in further study [33]. Then, Wilconson’s rank sum test was used to compare 22 types of immune cells between the high-risk and low-risk groups.

## Results

### Baseline

According to the criteria, 255 soft tissue sarcoma patients were selected in our research. Then, 170 patients were incorporated into the training set and the remaining 85 patients were used to form the testing set. The baseline information of all patients shown in Table 1. The results indicated that the differences of clinical data between the training and testing sets were not statistically significant.

### Identification of prognostic IRGs in sarcoma patients

**Fig. 1:**
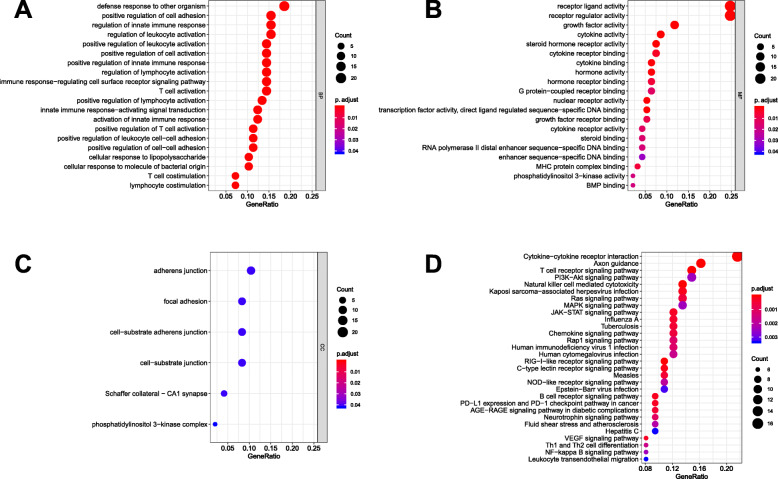
The enrichment analysis of overall survival-related immune-related genes in sarcoma patients. **a-c** The top 20(if possible) most significant Gene ontology analysis; **a**. biological process, **b**. molecular function, **c**. cellular component. **d**. The top 20 most significant Kyoto Encyclopedia of Genes and Genomes pathways

To identify the prognostic value of IRGs in soft tissue sarcoma patients, univariate Cox proportional hazard model was performed in 170 patients. Totally, 105 IRGs were selected as OS-related IRGs (Supplementary 1). Moreover, GO and KEGG enrichment analyses were performed, and the results are shown in Fig. 1, which indicated that the major enriched GO terms of BP were defense response to other organism, positive regulation of cell adhesion, regulation of innate immune response, regulation of leukocyte activation, and positive regulation of leukocyte activation. In CC, the major enriched GO terms were adherens junction, focal adhesion, cell−substrate adherens junction, cell−substrate junction and Schaffer collateral − CA1 synapse. For MF, we can find that the OS-related IRGs were mainly enriched in receptor ligand activity, receptor regulator activity, growth factor activity, cytokine activity, and steroid hormone receptor activity. For KEGG pathway analysis, it showed that many immune- or tumor-related pathways were identified, such as T cell receptor signaling pathway, Natural killer cell mediated cytotoxicity, Kaposi sarcoma−associated herpesvirus infection, PD − L1 expression and PD − 1 checkpoint pathway in cancer, Th1 and Th2 cell differentiation, and NF − kappa B signaling pathway.

### Construction of a TF regulatory network

**Fig. 2:**
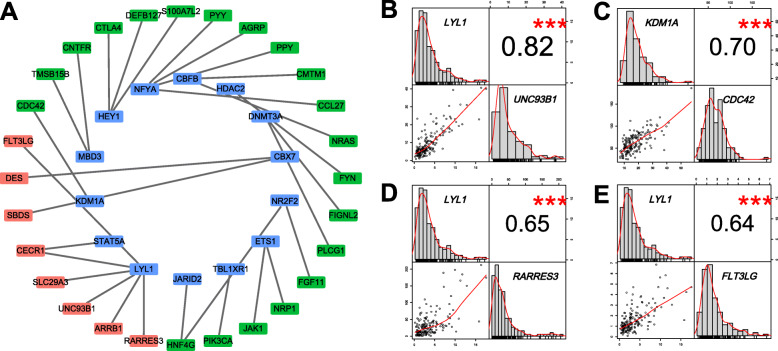
Transcription factors-based regulatory network. **a**. Regulatory network of prognostic transcription factors and prognostic immune-related genes. The rectangle represent prognostic transcription factors, the green rectangle represent poor prognosis-related immune-related genes, and the red rectangle represent good prognosis-related immune-related genes. **b-e**. Four examples to show the distribution of prognostic transcription factors and prognostic immune-related genes

To elucidate regulatory mechanisms of OS-related IRGs, we used univariate Cox proportional hazard model to identify the OS-related TFs. Totally, 36 OS-related TFs were confirmed (Supplementary 2). Furthermore, we analyzed the correlation between the expression of OS-related TFs and OS-related IRGs, the correlation results are shown in Supplementary 3. Interestingly, the results indicated that all TFs were positively correlated with IRGs. To better illustrate the regulatory relationship between TFs and IRGs, a TF-based regulatory network was generated (Fig. 2).

### Construction development and validation of the IRGs prognostic signature in training cohort

**Fig. 3:**
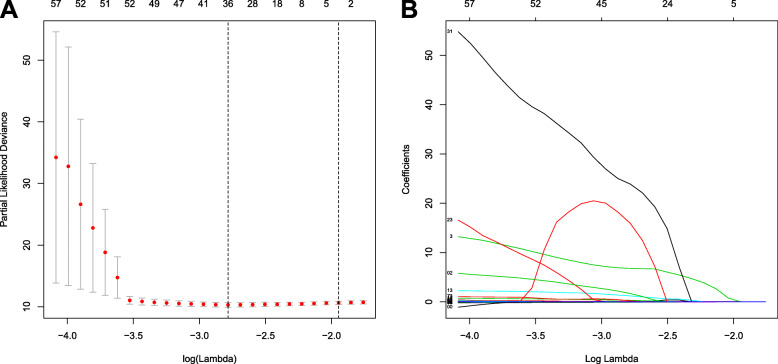
LASSO regression to select the most significant prognosis-related immune-related genes. LASSO: least absolute shrinkage and selection operator

**Fig. 4:**
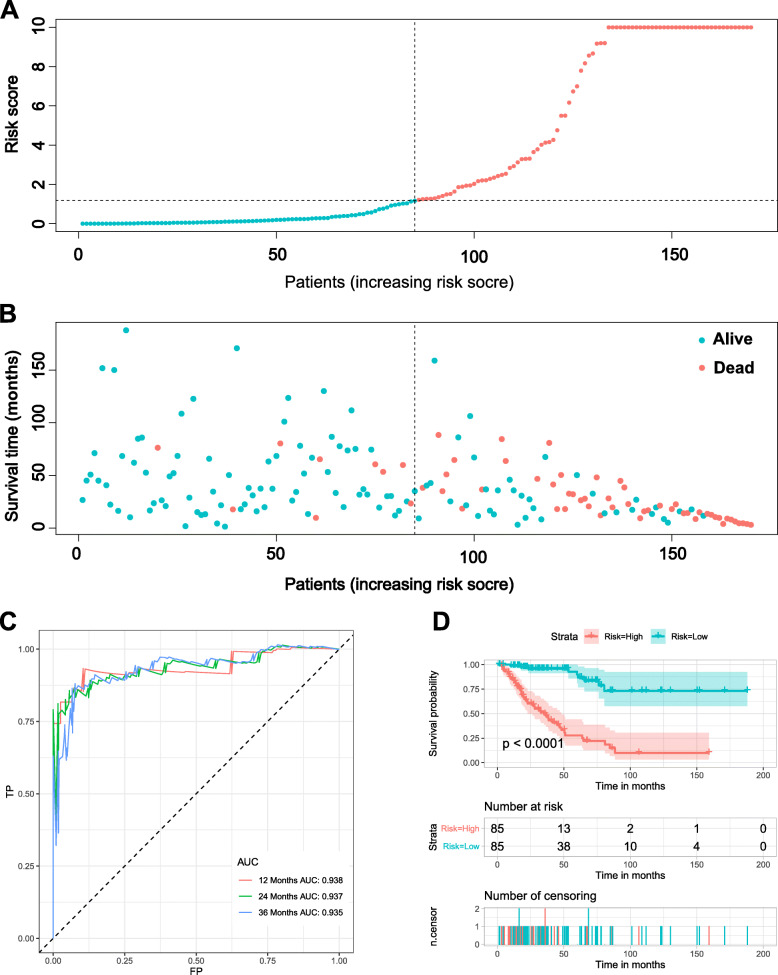
Construction of an immune-related prognostic signature in the training cohort. **a**. The risk score distribution of patients in the training cohort. **b**. Survival status scatter plots for patients in the training cohort. **c**. Time-dependent ROC curve analysis of the immune-related prognostic signature. **d**. Kaplan-Meier of overall survival in high- and low-risk group of the sarcoma patients in the training cohort

Based on 105 OS-related IRGs, the LASSO regression was used to choose the best appropriate genes as the prognostic predictors of the model. Thirty-five genes were selected in the LASSO regression analysis (Fig. 3). Then, the multivariate Cox proportional hazard model was performed based on the significant genes in the LASSO analysis, and a prognostic signature was established based on the 19 prognostic IRGs (Supplementary 4 and Fig. 4). The time-dependent ROC of 1-, 2-, and 3-years were shown in Fig. 4c. The AUC values of 1-, 2-, and 3-years were 0.938, 0.937, and 0.935, respectively, which means that the prognostic signature can serve as a valid tool for prognostic prediction in sarcoma patients (Fig. 4c). In addition, the risk scores of each patient in the training set were calculated, the median of risk score was used as the cutoff to stratify patients into high-risk (*n* = 85) and low-risk (*n* = 85) groups. The survival curve was generated, and the log-rank test indicated that the patients in the low-risk group had a favorable prognosis (Fig. 4d).

### External validation of IRGs signature

**Fig. 5:**
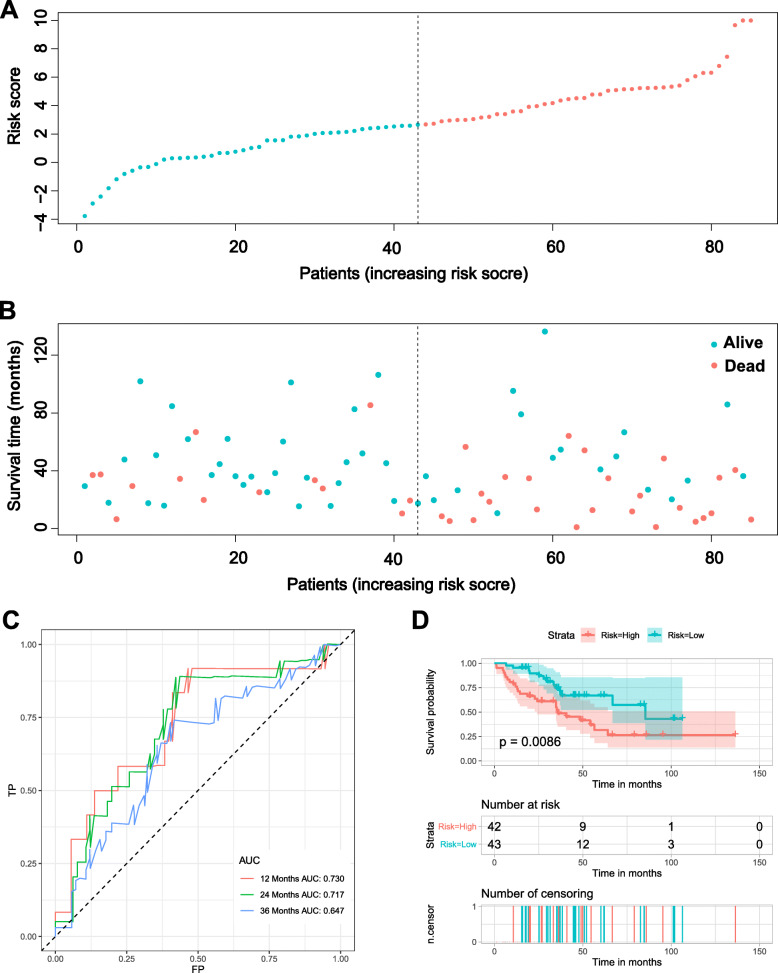
Validation of immune-related prognostic signature in the testing cohort. **a**. The risk score distribution of patients in the testing cohort. **b**. Survival status scatter plots for patients in the testing cohort. **c**. Time-dependent ROC curve analysis of the immune-related prognostic signature. **d**. Kaplan-Meier of overall survival in high- and low-risk group of the sarcoma patients in testing cohort

To further verify the stability and reliability of the risk signature based on the IRGs, an independent set was used. As the formula of risk score in the training set, the risk scores of each patient sample in the testing set were calculated (Fig. 5). The time-dependent ROC curves were generated to test the discrimination of the signature (Fig. 5c). The results showed that the AUC values of 1-,2-, and 3-year were 0.730, 0.717, and 0.647, which also showed good accuracy of nomogram in predicting the OS of sarcoma patients. Furthermore, according to the median of the risk score in the testing set, 85 patients were stratified into the low-risk group (*n* = 43) and high-risk group (*n* = 42). The survival curve of two groups was generated, and the results indicated that patients in the high-risk group have a worse prognosis (Fig. 5d).

### Development of a nomogram based on the IRG signature and clinical data

**Fig. 6:**
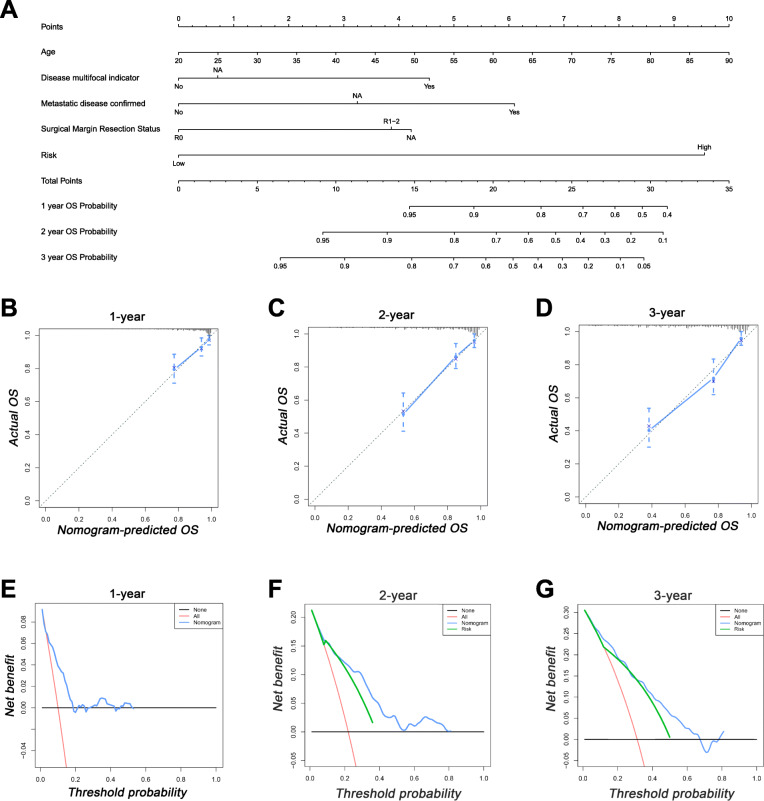
A nomogram based on immune-related prognostic signature and prognostic clinical variables. **a**. A nomogram for the prediction of the prognosis of sarcoma patients at 1, 2, and 3 years. **b-d**. Calibration curves of nomogram. **e-g**. Decision curve analysis of nomogram

To further construct a prognostic nomogram combining IRGs and clinical data, we performed univariate and multivariate Cox regression analysis to assess the independent prognostic variables for soft tissue sarcoma patients. In the univariate analysis, age, disease multifocal indicator, metastatic disease confirmed, surgical margin resection status and risk score were associated with the prognosis of sarcoma patients (Table 2). Then, the significant variables in the univariate Cox analysis were incorporated into the multivariate Cox analysis, and five independent prognostic variables were identified (Table 2). According to the results of the multivariate Cox analysis, we can find that risk score has the greatest impact for OS. In addition, higher age, multifocal sarcoma, tumor metastasis, and surgical resection status (R1–2) were also associated with worse prognosis in soft tissue sarcoma patients (Table 2). To better predict the prognosis of soft tissue sarcoma patients, we constructed a nomogram based on the independent factors determined in the multivariate regression (Fig. 6a). The C-index of our nomogram was 0.775 (95%CI:0.751–0.799), which showed good accuracy in predicting the prognosis of soft tissue sarcoma patients. The favorable calibration plot of our nomogram indicated that the OS predicted by the nomogram is highly consistent with the actual observation (Fig. 6b-d). In addition, DCA was also performed, and the results indicated the nomogram can serve as an effective prognostic model for soft tissue sarcoma patients (Fig. 6e-g).

**Table 2:** Univariate and multivariate Cox analysis in sarcoma patients

	Univariate analysis				Multivariate analysis			
	HR	95%CI		*P*	HR	95%CI		*P*
Age	1.020	1.004	1.036	0.012	1.025	1.008	1.043	0.004
Sex								
Female								
Male	0.849	0.565	1.274	0.429				
Race								
Asian				0.412				
African American	1.108	0.135	9.098	0.924				
WHITE	0.810	0.111	5.907	0.836				
Unknown	2.004	0.207	19.378	0.548				
Histological type								
DLP				0.903				
LMS	0.855	0.515	1.419	0.543				
MYX	0.730	0.339	1.571	0.421				
UPS	0.960	0.509	1.810	0.899				
Other	0.746	0.321	1.734	0.496				
Multifocal indicator								
No				0.002				0.008
Yes	2.328	1.443	3.754	0.001	2.228	1.324	3.748	0.003
Unknown	1.220	0.606	2.459	0.577	1.135	0.486	2.651	0.770
Metastasis								
No				< 0.001				< 0.001
Yes	2.962	1.797	4.880	< 0.001	2.926	1.721	4.972	< 0.001
Unknown	1.795	1.078	2.987	0.024	1.770	1.005	3.115	0.048
Radiotherapy								
No				0.963				
Yes	1.012	0.633	1.620	0.959				
Unknown	1.075	0.638	1.812	0.786				
Surgical margin resection status								
R0				< 0.001				0.005
R1–2	2.418	1.572	3.719	< 0.001	1.974	1.257	3.099	0.003
Unknown	2.194	1.156	4.165	0.016	2.099	0.960	4.589	0.063
Tumor site								
Extremity				0.745				
Other	1.182	0.699	1.997	0.533				
Retroperitoneum/Upper abdominal	1.193	0.735	1.934	0.475				
Risk	5.192	3.215	8.382	< 0.001	5.362	3.241	8.868	< 0.001

*LMS* Leiomyosarcoma, *DLP* Dedifferentiated liposarcoma, *UPS* Undifferentiated pleomorphic sarcoma, *MYX* Myxofibrosarcoma

### Subgroup analyses of nomogram

**Fig. 7:**
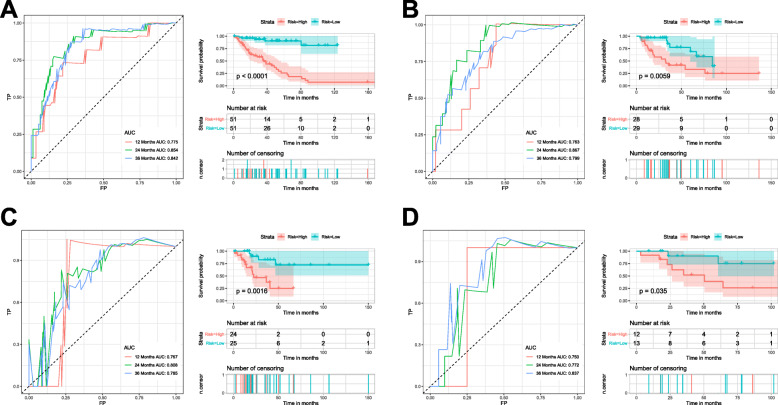
Subgroup analysis of nomogram. **a**. The ROC curve and Kaplan-Meier curve to show the prognostic value of nomogram in leiomyosarcoma. **b**. The ROC curve and Kaplan-Meier curve to show the prognostic value of nomogram in dedifferentiated liposarcoma. **c**. The ROC curve and Kaplan-Meier curve to show the prognostic value of nomogram in undifferentiated pleomorphic sarcoma. **d**. The ROC curve and Kaplan-Meier curve to show the prognostic value of nomogram in myxofibrosarcoma

To further confirm that the nomogram can perform stably in different histological types of soft tissue sarcoma, patients were divided into different subgroups (Fig. 7). We can find the AUC values of the nomogram were higher than 0.750(range:0.750–0.867) in all subgroups, which means that the nomogram can perform stably in different histological types of soft tissue sarcoma. In addition, in all subgroups, the Kaplan-Meier survival curves and the log-rank test indicated that the patients in the high-risk group have a poorer prognosis than patients in the low-risk group (all *p* < 0.05) (Fig. 7).

### Comparison of the immune infiltration cell in low- and high-risk group

**Fig. 8:**
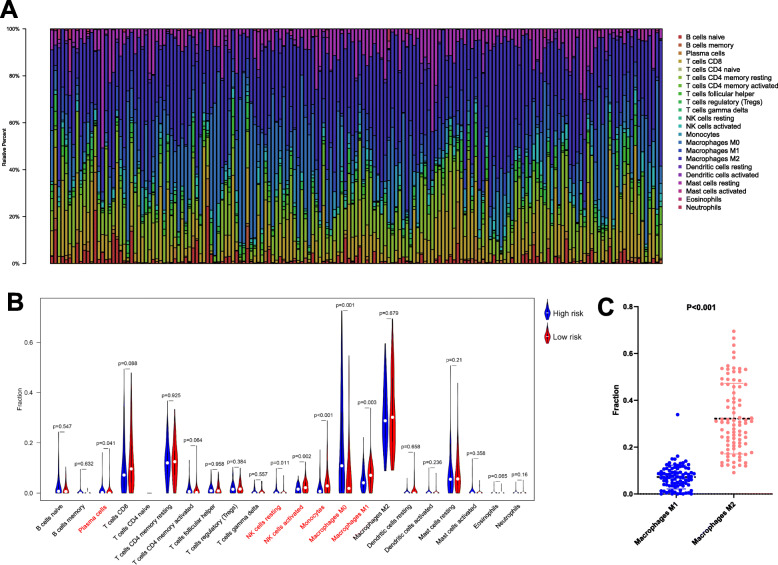
Immune infiltration status of immune-related risk groups. **a**. The distribution of 22 immune cells in 169 sarcoma patients. **b**. Comparison of immune infiltration cell between high- and low-risk group. **c**. The infiltration of macrophages including M1 and M2 in the low-risk groups

After CIBERSORT package was performed, 169 patients with complete data of immune infiltration cells were included in this part of the study (Fig. 8a). Among the 169 patients, 87 were in the low-risk group and 82 were in the high-risk group. The results showed that six immune cells were significantly different between the two groups (Fig. 8b). The infiltration level of plasma cells and macrophages M0 were significantly higher in the high-risk group, while the infiltration level of NK cells resting, NK cells activated, monocytes, and macrophages M1 were significantly higher in the low-risk group (Fig. 8b). Additionally, as macrophage plays a key role in influencing the anti-tumor immune responses, we further analyzed the infiltration of macrophages M1 and M2 in the low-risk groups. The results showed that the infiltration level of macrophages M2 were significantly higher than the infiltration level of macrophages M1 (Fig. 8c).

## Discussion

Due to the heterogeneity and high metastatic rate of sarcoma, treatment methods were very limited and the outcome was unfavorable. Therefore, it is necessary for us to identify the effective biomarkers for the prognosis of sarcoma patients. In the present study, 105 IRGs were identified as prognostic IRGs and the regulatory network between prognostic IRGs and TFs was established. More importantly, we constructed a prognostic signature based on 19 IRGs and validated its effectiveness, which also was proved to be associated with immune infiltration cells. Furthermore, a nomogram incorporating IRG-based signature and clinical data was established, which showed excellent performance to predict the outcome of sarcoma patients.

With the wide exploration of immune mechanisms in the pathogenesis and progression of the tumor, many IRGs were identified as prognostic biomarkers in tumor patients [17–20, 23–26, 34]. More importantly, based on the researches of tumor immunology, many immunotherapies have been developed and become new methods for treating sarcoma patients, and results indicated that the outcome was satisfactory [35–37]. However, as far as we know, it is the first research to establish a signature based on IRGs for sarcoma patients, which may open up a novel perspective for constructing an effective prognostic model and improving sarcoma patient management of immunotherapy.

In our research, a prognostic signature incorporating 19 IRGs was established. The AUC of the model was higher than 0.9 in the training set, which showed great accuracy in predicting the prognosis of sarcoma patients. Moreover, the accuracy of our model was also successfully verified by an independent set. Our results indicated that the model can be used to identify sarcoma patients at high risk and enabling early interventions to improve the prognosis. Among 19 IRGs enrolled in risk signature, VEGFA, CYR61, and RHOA have confirmed to be related to the pathogenesis or prognosis of sarcoma [38–41]. It was reported that VEGFA is a potential neovascular regulatory factor that promotes tumor growth and metastasis through its receptor. Differential expression of VEGFA subtypes regulates sarcoma metastasis and response to anti-VEGFA [38]. Besides, the VEGF family can not only promote tumor-associated immunodeficiency by interfering with the growth of early hematopoietic progenitor T cells, but promote Treg cell proliferation [42, 43]. CYR61 is a member of the CCN family. The expression level of CYR61 is associated with the aggressiveness of osteosarcoma in different pre-clinical models and patient tumor samples, and CYR61 triggers osteosarcoma metastasis and spread through an IGF1Rβ-dependent EMT-like process [44]. The metastasis of invasive amoebic sarcoma cells depends on the Rho / Rock / MLC signal, and RHOA overexpression is related to tumor cell invasion and migration [41]. The relationship between the remaining 16 genes and sarcoma has few been reported, however, most of them have been confirmed to be associated with other tumors, which suggested that these genes also play essential roles in sarcoma. For example, PSMD10 is a crucial oncoprotein that is up-regulated in a variety of cancers and has potential in the initiation and progression of tumors [45]. Studies have indicated important roles played by PSMD10 in the pathogenesis of liver cancers and colorectal cancers, and PSMD 10 involved in regulating the proliferation and metastasis of thyroid cancers [46].

Combining the results of the above KEGG pathway and immune genes revealed the existence of potential immune mechanisms in sarcoma. Previous studies have suggested that high levels of macrophage infiltration predict a poor prognosis for Ewing’s sarcoma, which is consistent with the result that macrophage M0 significantly increased in high-risk groups in our study [47]. Additionally, different subtypes of macrophage showed different infiltration model and may serve as different role in cancer progression and anti-tumor immune responses. Macrophage M1 not only associated with the OS of sarcoma, but also significantly associated with recurrence of sarcoma [48]. macrophages M2 were found to be the most abundant immune cell type and were associated with improved survival in osteosarcoma [49]. Interestingly, Dhupkar et.al indicated that osteosarcoma lung metastases regression by anti-PD1 can be attributed to activated tumor macrophages M1 and reduced macrophages M2 [50]. Although there are few studies focused on macrophages M0, its relationship with other immune cells, such as CD8 T cells, has also been preliminarily reported [51]. Natural killer (NK) cells are important immune cells in the innate immune mechanisms. The activation of NK cells can promote the immunotherapy of Ewing’s sarcoma [52]. In this study, we found that activated NK cells were higher in the low-risk group and enriched in the KEGG pathway, which can provide a theoretical basis for immunotherapy in sarcoma patients.

In addition, in order to make the clinical application more convenient and accurate, a nomogram was established by combining immune signature and clinical data. The results of the AUC, calibration curve, DCA and subgroup analysis indicated that the nomogram can serve as an effective tool for predicting the prognosis in sarcoma patients. Although a lot of predictive models for sarcoma patients were constructed based on the clinicopathologic data, lncRNA, plasmacytoma variant translocation 1 and other predictors [13, 53, 54]. However, it should point out that the discriminative ability of the previous models was low with AUC or C-statistic less than 0.750 [13, 53–55], which means that they are unsatisfactory. Therefore, we think that the IRG-based nomogram can improve the risk stratification for sarcoma patients.

There are some limitations to our research. Firstly, this is a retrospective study, which may lead to bias and a novel sight and large cohort to verify is needed. Second, although we find the prognostic ability of the signature and develop a nomogram with great predictive ability, more clinical features are needed to improve the nomograms, such as AJCC TNM stage. Thirdly, this study was a bioinformatic study based on a public database, and the experiment should be performed in the future to explore the mechanism of the effects of IRGs in the prognosis of sarcoma. Finally, the training and testing sets were essentially from one cohort and the prognostic signature has no real external validation. Therefore, the validation of this prognostic model was relative weak and further validation in independent cohort is needed.

## Conclusion

In summary, we identified and validated an IRG-based signature, which can be used as an independent prognostic signature in evaluating the prognosis of sarcoma patients. However, further experimental exploration is needed to study the potential mechanism of IRGs in sarcoma.

## Supplementary Information

**Additional file 1.**

**Additional file 2.**

**Additional file 3.**

**Additional file 4.**

## Data Availability

The data of this study are from The Cancer Genome Atlas (https://portal.gdc.cancer.gov/) and ImmPort database (https://www.immport.org/).

## Abbreviations

IRGs: Immune-related genes
TCGA: The Cancer Genome Atlas
ROC: Receiver operating characteristic
AUC: Area under the curve
RNA-seq: RNA-sequencing
OS: Overall survival
GO: Gene Ontology
MF: Molecular function
BP: Biological process
CC: Cellular component
KEGG: Kyoto Encyclopedia of Genes and Genomes
K-M: Kaplan-Meier
DCA: Decision curve analysis
NK: Natural killer
BCR: B cell antigen receptor
PD1: Programmed cell death 1
PDL-1: Programmed Death Ligand-1
